# Development and Validation of Analytical Procedure for Elemental Impurities in Rosuvastatin Calcium Tablets by ICP-MS and Microwave Digestion

**DOI:** 10.1155/2024/9952318

**Published:** 2024-03-26

**Authors:** Yajie Hao, Guang Yin, Xuemei Wang, Juan Fu, Qihui Cao, Qinyong Sun, Guimin Zhang, Zhong Feng

**Affiliations:** ^1^National Engineering Research Center of Chiral Drugs, Lunan Pharmaceutical Group, 1 North Outer Ring Road, Fei, Linyi, Shandong, China; ^2^School of Pharmaceutical Sciences (Shenzhen), Sun Yat-sen University, Shenzhen, China

## Abstract

Rosuvastatin calcium is a widely used 3-hydroxy-3-methylglutaryl coenzyme A-reductase inhibitor developed for the treatment of dyslipidemia. To establish a control strategy for the elemental impurities, a new digestion method combined with an inductively coupled plasma-mass spectrometer (ICP-MS) was developed and validated by our team to determine elements Cd, Pb, As, Hg, Co, V, and Ni in rosuvastatin calcium tablets, which digest the sample perfectly even in the presence of a large number of excipients, especially titanium dioxide. The measurement mode was collision cell mode with kinetic energy discrimination (KED). 209Bi^+^, 115In^+^, and 89Y^+^ were chosen as internal standard elements. The recoveries of the limit of quantitation (LOQ) ranged from 90.5% to 106.4%, concentrations of the abovementioned elements in LOQ were 0.25 *µ*g·L^−1^, 0.25 *µ*g·L^−1^, 0.75 *µ*g·L^−1^, 1.5 *µ*g·L^−1^, 2.5 *µ*g·L^−1^, 5 *µ*g·L^−1^, and 8 *µ*g·L^−1^ , respectively, linear correlation coefficients were above 0.9997, the recoveries in accuracy item ranged from 91.8% to 103.6%, and relative standard deviations (RSDs) of recovery in precision were not more than 1.8%, reflecting a reliable method of high sensitivity, strong anti-interference capacity, and good precision, and that it was suitable for the determination of elemental impurities in drugs.

## 1. Introduction

Rosuvastatin calcium tablets, which served as an oral 3-hydroxy-3-methylglutaryl coenzyme A-reductase inhibitor, were extensively employed for the treatment of dyslipidemia, especially in elderly patients with coronary heart disease complicated by hyperlipidemia [1, 2]. Given that hypolipidemic drugs need to be prescribed lifelong for most of the selected patients [3], much more importance should be attached to the control of elements in rosuvastatin calcium tablets. According to USP general chapter 232, risk assessment for Cd, Pb, As, Hg, Co, V, and Ni that were listed in class 1 and class 2A was required, which should be focused on assessing the levels of elemental impurities in tablets in relation to the permissible daily exposures (PDEs) presented in USP 232 and ICH Q3D [4, 5]. V was considered to be a human carcinogen that was of genetic toxicity but not mutagenic; multiple oral doses of Co could cause human erythrocytosis; exposure to Pb resulted in harmful effects on the central nervous system, reproductive system, and immune system; and Hg absorbed via the respiratory system would damage the brain [6–10]. Thus, a mature analytical procedure for the 7 elemental impurities is indispensable.

Microwave digestion combined with ICP-MS was commonly used for the analysis of elemental impurities in foodstuffs, active pharmaceutical ingredients, plants, as well as crude oil [11–17], because of its higher sensitivity than inductively coupled plasma optical emission spectrometry and atomic absorption spectrometry. There are several reports on elemental impurities in pharmaceutical tablets. However, taking the compatibility of digestion parameters with the instrument into account, the digestion methods that used sulfuric acid or employed high temperature (450°C) were not adopted in this study [18–20]. During the preparation process of rosuvastatin calcium tablets, microcrystalline cellulose, lactose monohydrate, calcium phosphate, cross-linked povidone, magnesium stearate, and titanium dioxide were employed as excipients, which bring great difficulties in sample digestion because of their specialties of stability and firmness. For example, titanium dioxide is very thermally stable and extremely resistant to chemical degradation [21].

A new efficient microwave digestion method combined with ICP-MS was proposed and validated based on the preparation process of rosuvastatin calcium tablets and requirements of the USP 232 chapter and ICH Q3D guideline by our team, where sulfuric acid or high temperature (450°C) was avoided to digest complex samples and volatilization of Hg was reduced. Limits of elemental impurities of Cd, Pb, As, Hg, Co, V, and Ni were 0.5, 0.5, 1.5, 3, 5, 10, and 20 ppm, respectively. Concentrations of these elements in solution were 1 *µ*g·L^−1^, 1 *µ*g·L^−1^, 3 *µ*g·L^−1^, 6 *µ*g·L^−1^, 10 *µ*g·L^−1^, 20 *µ*g·L^−1^, and 40 *µ*g·L^−1^, respectively.

## 2. Materials and Methods

### 2.1. Materials

Apparatus: The Milli-Q Integral 10 system (Merck, Germany) was used to provide ultrapure water. The graphite furnace (Labotery, China), ETHOS UP microwave digestion apparatus with MAXI-44 high-throughput rotor (Milestone, Italy), and Mettler Toledo XS204 balance (Switzerland) were employed during the preparation of solutions. The iCAP RQ mass spectrometer (ICP-MS, Thermo Fisher Scientific, USA) equipped with ASX-560 autosampler (Teledyne CETAC Technologies) was used to detect elements.

Reagents: Optima grade concentrated nitric acid (Fisher Scientific Chemical, USA), guaranteed reagent hydrofluoric acid (Aladdin, USA), guaranteed reagent hydrochloric acid (Sinopharm Chemical Reagent Co., Ltd., China), analytical reagent concentrated sulfuric acid (Xilong Scientific, China), and analytical Reagent 30% (m/m) hydrogen peroxide (Xilong Scientific, China) were used to prepare solutions. Argon (99.999%) used as a cooling gas, nebulizer gas, and auxiliary gas and Helium (99.999%) used as a collision gas were obtained from Jinan Deyang Special Gas Co., LTD (Shandong, China).

Standards: standards used were as follows: ICP Multi-Element standard (Reagecon, Ireland) containing 5 elements, Bi, In, Sc, Tb, and Y at 100 mg·L^−1^ in 2% (v/v) nitric acid; ICP Multi-Element standard (Reagecon, Ireland), 24 elements, bottle 1 of 3, containing 5 *µ*g·mL^−1^ cadmium, 5 *µ*g·mL^−1^ lead, 15 *µ*g·mL^−1^ arsenic, 30 *µ*g·mL^−1^ mercury, 50 *µ*g·mL^−1^ cobalt, 100 *µ*g·mL^−1^ vanadium, and 200 *µ*g·mL^−1^ nickel; and 100 *µ*g·mL^−1^ of ICP standard Gold (Reagecon, Ireland). Rosuvastatin calcium tablets were obtained from (batch nos. 0009190301, 0009190302, and 0009190303) Lunan Pharmaceutical Group, Shandong, China.

### 2.2. Methods

#### 2.2.1. Digestion Procedure

Sample digestion was performed by using a microwave digestion system whose parameters are listed in Table 1.

#### 2.2.2. Parameters of ICP-MS

All detections were carried out with ICP-MS, whose parameters are described in Table 2 and Supplementary file (available here). The most abundant isotopes of 7 elements, 111Cd^+^, 208Pb^+^, 75As^+^, 202Hg^+^, 59Co^+^, 51V^+^, and 60Ni^+^, were selected for quantification and determined in KED mode. Internal standards 209Bi^+^, 115In^+^, 45Sc^+^, and 89Y^+^ were also determined in KED mode.

#### 2.2.3. Preparation of Solutions

Concentrations of all samples were related to oral PDEs of elements presented in USP 232 and ICH Q3D that are listed in Table 3.

*Blank Solution.* 0.2 mL of ICP standard Gold was transferred to a 100 mL clean plastic volumetric flask, diluted to the volume with 2% (v/v) nitric acid, and mixed.

*Internal Standard Solution*. 37.5 *µ*L of ICP Multi-Element standard containing 5 elements was transferred to a 250 mL clean plastic volumetric flask, diluted to the volume with 2% (v/v) nitric acid, and mixed.

*Standard Stock Solution*. 1 mL of ICP Multi-Element standard containing 24 elements was transferred to a 10 mL clean plastic volumetric flask, diluted to the volume with 2% (v/v) nitric acid, and mixed.

*Standard Solutions*. 50 *µ*L, 100 *µ*L, 200 *µ*L, 300 *µ*L, and 500 *µ*L of standard stock solution were transferred to five 100 mL clean plastic volumetric flasks separately. 0.2 mL of ICP standard Gold was transferred to the abovementioned flasks, respectively. The mixture was diluted to the volume with 2% (v/v) nitric acid and mixed. Solutions were named as L1, L2, L3, L4, and L5 accordingly. Concentrations of Cd in L1 to L5 ranged from 0.25 *µ*g·L^−1^ to 2.5 *µ*g·L^−1^, Pb ranged from 0.25 *µ*g·L^−1^ to 2.5 *µ*g·L^−1^, As ranged from 0.75 *µ*g·L^−1^ to 7.5 *µ*g·L^−1^, Hg ranged from 1.5 *µ*g·L^−1^ to 15 *µ*g·L^−1^, Co ranged from 2.5 *µ*g·L^−1^ to 25 *µ*g·L^−1^, V ranged from 5 *µ*g·L^−1^ to 50 *µ*g·L^−1^, and Ni ranged from 10 *µ*g·L^−1^ to 100 *µ*g·L^−1^.

*Sample Blank Solution*. 5 mL of nitric acid, 50 *µ*L of hydrofluoric acid, and 0.2 mL of ICP standard Gold were transferred to a digestion tank and mixed. The mixture was digested in the microwave digestion apparatus at the required parameters (Table 1). After that, the mixture was heated in a graphite furnace at 105°C till the liquid in the tank was nearly dried. The mixture left in the tank was transferred to a 100 mL plastic volumetric flask after cooling and diluted to volume with 2% (v/v) nitric acid and mixed.

*Sample Solution.* Rosuvastatin calcium tablets were ground into powder. The powder (0.2 g) was weighed and placed in a digestion tank. 5 mL of nitric acid, 50 *µ*L of hydrofluoric acid, and 0.2 mL of ICP standard Gold were added to the tank and mixed. The mixture was digested in a microwave digestion apparatus at the required parameters (Table 1). After that, the mixture was heated in a graphite furnace at 105°C till the liquid in the tank was nearly dried. The mixture in the tank was diluted by 2% (v/v) nitric acid after cooling and transferred to a 100 mL plastic volumetric flask, diluted to volume with 2% (v/v) nitric acid, and mixed.

Spiked samples are described as follows.

*Low Level*. The powder (0.2 g) was weighed accurately and placed in a digestion tank. 5 mL of nitric acid, 50 *µ*L of hydrofluoric acid, 0.2 mL of ICP standard Gold, and 100 *µ*L of standard stock solution were added into the tank. They were digested in a microwave digestion apparatus at the required parameters (Table 1) and then heated in a graphite furnace at 105°C till the liquid in the tank was nearly dried. After that, the mixture in the tank was diluted by 2% (v/v) nitric acid and transferred to a 100 mL plastic volumetric flask after cooling. Finally, the mixture was diluted to volume with 2% (v/v) nitric acid and mixed. Concentrations of 7 elements added in spiked samples were equal to that in the L2 solution.

The medium level was the same as the low level, except that 200 *µ*L of standard stock solution was added into the tank. Concentrations of 7 elements added in spiked samples were equal to that in the L3 solution.

The high level was the same as the low level, except that 300 *µ*L of standard stock solution was added into the tank. Concentrations of 7 elements added in spiked samples were equal to that in the L4 solution.

LOQ solution was the same as low level, except that 50 *µ*L of standard stock solution was added into the tank. The concentrations of 7 elements added in spiked samples were equal to that in the L1 solution.

#### 2.2.4. System Suitability Test

Standard solution L4 was injected into ICP-MS to test the system' suitability, at the beginning and after the other 9 runs of the sample. Elements intensities in the L4 standard solution run later were compared with that of the first injection.

#### 2.2.5. Linearity Test

Blank solution and standard solutions L1 to L5 were tested, and element intensities were recorded. After that, canonical plotting was drawn, in which concentration was the *x*-axis and intensity was the *y*-axis, to calculate the linear equation and *r* value.

#### 2.2.6. Specificity Test

The blank solution, sample blank solution, sample solution, and LOQ solution were tested. Element intensities were recorded. There should be no interference for the quantification of analytes in blank and sample blank solutions; that is, the ratios of analytes' response to internal standards' response in blank solutions should be less than that in LOQ solutions.

#### 2.2.7. Accuracy and LOQ Test

Sample blank solution, sample solution, triplicate LOQ solutions, triplicate low-level spiked samples, triplicate medium-level spiked samples, and triplicate high-level spiked samples were tested. Recoveries of elements in the spiked sample were calculated.

#### 2.2.8. Precision Test

Repeatability test: sample blank solution, sample solution, and six medium-level spiked samples were tested. Recoveries of elements as well as their RSDs in six spiked samples were calculated.

Intermediate precision test: blank solution, standard solutions L1 to L5, sample blank solution, sample solution, and six medium-level spiked samples were tested on another day. Recoveries of elements as well as their RSDs in six spiked samples were calculated. Moreover, the RSD of element recoveries in twelve spiked samples (repeatability and intermediate precision) was calculated.

The internal standard solution was injected into ICP-MS during all runs of the sample.

#### 2.2.9. Sample Testing

The blank solution, standard solutions L1 to L5, sample blank solution, and 3 batches of sample solution were tested. The elements' content in the rosuvastatin calcium tablet was calculated.

## 3. Results and Discussion

### 3.1. Exploration of Pretreatment Procedure of Samples

Rosuvastatin calcium tablets cannot be dissolved by water or any other organic solvent, and hence, a digestion method is required. Microwave digestion condition was confirmed after 10 trials that are shown in Table 4. A higher concentration of hydrofluoric acid would harm the environment, and a sampling system made of quartz and 5 mL of nitric acid plus 0.05 mL of hydrofluoric acid were suggested.

In addition, ICP standard Gold was used to guarantee that the recoveries of mercury in spiked samples were within the range of 70%–150% specified in the USP 233 chapter, as discussed by [22].

### 3.2. Selection of Isotopes and Internal Standard Elements

There are multiple isotopes in the majority of elements. In this study, isotopes with a better specificity and higher abundance ratio (higher sensitivity) of the 7 elements were selected, namely, 111Cd^+^, 208Pb^+^, 75As^+^, 202Hg^+^, 59Co^+^, 51V^+^, and 60Ni^+^, as shown in Table 5. The selected internal standard elements should not exist in the sample to be tested, and the mass number of internal standard elements was suggested to be close to that of the target elements, ensuring that the behavior of the internal standard elements and target elements was similar under one condition. Furthermore, the ionization potential of the internal standard element and target element should be close. Therefore, 89Y^+^, 115In^+^, and 209Bi^+^ were selected as the internal standard elements in the experiment.

### 3.3. Method Validation

System suitability, linearity, specificity, limit of quantitation, accuracy, and precision were studied according to the USP 233 chapter [5]. The results were all within the acceptance criteria, which showed a reliable method. The limits of analytes were determined by their PDEs and the maximum daily dose (MDD, 40 mg/d) of the drug product. According to option 1 in ICH Q3D guideline [4], common permitted concentration limits of elements across drug product components for drug products with daily intakes of not more than 10 grams were calculated by using the following equation. As a result, the limits of elemental impurities of Cd, Pb, As, Hg, Co, V, and Ni were 0.5, 0.5, 1.5, 3, 5, 10, and 20 ppm, respectively. The concentration of the sample (rosuvastatin calcium tablets) was confirmed as 2 mg·ml^−1^ after optimization of the digestion method. Accordingly, the concentrations of these elements in solution were 1 *µ*g·L^−1^, 1 *µ*g·L^−1^, 3 *µ*g·L^−1^, 6 *µ*g·L^−1^, 10 *µ*g·L^−1^, 20 *µ*g·L^−1^, and 40 *µ*g·L^−1^, respectively.

(1) $$\begin{array}{c} \text{Concentrations}\left(\text{ppm}\right)=\frac{\text{PDEs}\left(\mu g/d\right)}{10\left(g/d\right)}. \end{array}$$

**Figure 1:**
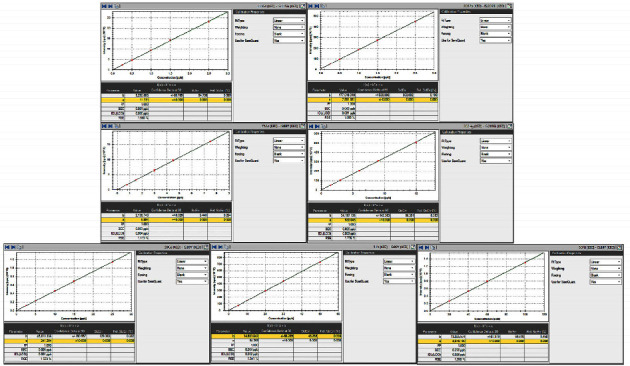
Linearity calibration plots of 7 elements.

During the system suitability test, the maximal drift value of 7 elements (Cd, Pb, As, Hg, Co, V, and Ni) was 6.7% and satisfied the specified system suitability parameters (NMT 20.0%), which showed that the ICP-MS system was reliable. Regression equations and LOQ are described in Table 6, and canonical plottings of elements are shown in Figure 1, which indicated that the method was of good linearity and a reasonably high sensitivity. Correlation coefficients (*r*) of Cd, Pb, As, Hg, Co, V, and Ni were 0.9998, 1.0000, 0.9999, 0.9999, 0.9999, 0.9999, and 0.9999, respectively, all above 0.99 as required in USP chapter 736 [23]. The ratios of elements to be tested and internal standard in blank and sample blank were lower than that of the LOQ solution, indicating that the analytical procedure was of great specificity, as illustrated in Table 7. Accuracy and precision of the method were reflected via recoveries of elements in spiked samples that ranged from 91.8% to 103.6% as well as RSDs that were not more than 2.0%, as presented in Tables 8 and 9, suggesting that this method was considerably accurate and precise. Recoveries were within the range of 70%∼150%, and RSDs were not more than 20.0% as required in USP chapter 233 [5]. Matrix effects on analytes were corrected by internal standards. Furthermore, recoveries of elements in spiked samples (close to 100%) showed that the matrix effect was reduced.

### 3.4. Elements Content in Tablet

The result showed that 7 elements in 3 process validation batches of tablets were not more than LOQ, namely, contents of 7 elements were lower than the control threshold regulated in ICH Q3D. Thus, additional controls were not required for elements in rosuvastatin calcium tablets.

The validated method indicated that a digestion system containing 5 mL of nitric acid and 0.05 mL of hydrofluoric acid was able to digest rosuvastatin calcium tablets composed of microcrystalline cellulose, lactose monohydrate, calcium phosphate, cross-linked povidone, magnesium stearate, and titanium dioxide at 190°C (a lower temperature than 450°C). Recoveries of Hg were all above 90% showing that the volatilization of mercury could be negligible at the condition of drying temperature of acid not higher than 105°C as well as at the condition of adding element gold into the digestion system.

## 4. Conclusions

The developed procedure, microwave digestion combined with the ICP-MS system, for quantitative elemental impurities measurement in rosuvastatin calcium tablets and the linearity, specificity, precision, and accuracy found according to ICH guidelines as well as USP 233 and 736 chapters have been validated. The selection of isotopes matters in the development of the method. In addition, hydrofluoric acid plays an important role in sample digestion, particularly for the drug product.

Moreover, the procedure was effective in regard to the existence of microcrystalline cellulose, lactose monohydrate, calcium phosphate, cross-linked povidone, magnesium stearate, and titanium dioxide in the sample, allowing this analytical approach for elemental impurities quantification in pharmaceutic preparation to be used efficiently and conveniently. However, to protect the sampling system of ICP-MS, the hydrofluoric acid used must be reduced as much as possible before injecting samples into the instrument. Hence, the mixture after being digested in a microwave digestion system was supposed to be heated in a graphite furnace at 105°C till the liquid in the tank was nearly dried, which extended the time of analysis.

## Figures and Tables

**Table 1:** The parameter of microwave digestion.

Sample mass (g)	Reagents added	Initial temperature (°C)	Ramp (min)	Target temperature (°C)	Hold (min)
0.2	5 mL of nitric acid, 50 *µ*L of hydrofluoric acid, 0.2 mL of ICP standard Gold	Room temperature	10	120	2
		120	5	150	5
		150	6	190	55

**Table 2:** The parameter of ICP-MS.

Parameters	Setup
Nebulization temperature (°C)	2.7
Peristaltic pump speed (r·min^−1^)	40
Nebulizer gas flow rate (L·min^−1^)	0.9964
Cooling gas flow rate (L·min^−1^)	14
Auxiliary gas flow rate (L·min^−1^)	0.8
Sampling depth (mm)	5
Collision gas flow rate (mL·min^−1^)	5.4
Plasma power	1550
CCT focus lens	3.6
Scan mode	KED
Number of scans	20
Time per scan (s)	0.5
Time of dwell (s)	0.05
Time of uptake (s)	45
Time of wash (s)	30
Torch vertical position	0.77
Extraction lens 2	−200.7

**Table 3:** Permitted daily exposures for elemental impurities established in ICH Q3D.

Element	Class	Oral PDE (*µ*g/day)	Parenteral PDE (*µ*g/day)	Inhalation PDE (*µ*g/day)
Cd	1	5	2	3
Pb	1	5	5	5
As	1	15	15	2
Hg	1	30	3	1
Co	2A	50	5	3
V	2A	100	10	1
Ni	2A	200	20	5
Tl	2B	8	8	8
Au	2B	100	100	1
Pd	2B	100	10	1
Ir	2B	100	10	1
Os	2B	100	10	1
Rh	2B	100	10	1
Ru	2B	100	10	1
Se	2B	150	80	130
Ag	2B	150	10	7
Pt	2B	100	10	1
Li	3	550	250	25
Sb	3	1200	90	20
Ba	3	1400	700	300
Mo	3	3000	1500	10
Cu	3	3000	300	30
Sn	3	6000	600	60
Cr	3	11000	1100	3

**Table 4:** Digestion procedure optimization.

	Acid added into the digestion tank	Mixture's state after digestion	Mixture's final state after dilution
1	5 mL of nitric acid	Muddy	Muddy
2	10 mL of nitric acid	Muddy	Muddy
3	9 mL of nitric acid plus 3 mL of hydrochloric acid	Muddy	Muddy
4	10 mL of nitric acid plus 2 mL of 30% hydrogen peroxide	Muddy	Muddy
5	5 mL of nitric acid plus 1 mL of concentrated sulfuric acid	Muddy	Muddy
6	5 mL of nitric acid plus 0.04 mL of hydrofluoric acid	Clear	Muddy
7	5 mL of nitric acid plus 0.05 mL of hydrofluoric acid	Clear	Clear
8	5 mL of nitric acid plus 0.06 mL of hydrofluoric acid	Clear	Clear
9	5 mL of nitric acid plus 0.1 mL of hydrofluoric acid	Clear	Clear
10	5 mL of nitric acid plus 0.5 mL of hydrofluoric acid	Clear	Clear

**Table 5:** Selection of elements' isotopes.

Element	Isotopes abundance ratio	Potential interference	Selected
Cd	114Cd (28.73%)	114Cd: 40Ar + 74Ge, 12C + 102Ru, 16O + 98Mo	111Cd
	112Cd (24.13%)	112Cd: 40Ar + 72Ge, 14N + 98Mo, 16O + 96Mo	
	111Cd (12.80%)		

Pb	208Pb (52.40%)	None	208Pb
	206Pb (24.10%)		
	207Pb (22.10%)		

As	75As (100%)	None	75As

Hg	202Hg (29.80%)	None	202Hg
	200Hg (23.13%)		
	199Hg (16.84%)		

Co	59Co (100%)	None	59Co

V	51V (99.75%), 50V (0.25%)	None	51V

Ni	58Ni (68.27%)	58Ni: 58Fe, 40Ar + 18O	60Ni
	60Ni (26.10%)		
	61Ni (1.13%)		

**Table 6:** Results of linearity, range, and LOQ.

Element	Range	Equations	*R* ^2^	LOQ (*µ*g L^−1^)
^111^Cd	0∼2.5 *µ*g·L^−1^	*f* (*x*) = 9292.8026*x* + 11.3308	0.9996	0.25
^208^Pb	0∼2.5 *µ*g·L^−1^	*f* (*x*) = 177518.3179*x* + 7591.9806	1.0000	0.25
^75^As	0∼7.5 *µ*g·L^−1^	*f* (*x*) = 2139.7435*x* + 6.9935	0.9999	0.75
^202^Hg	0∼15 *µ*g·L^−1^	*f* (*x*) = 34197.1263*x* + 688.0450	0.9999	1.5
^59^Co	0∼25 *µ*g·L^−1^	*f* (*x*) = 45810.3360*x* + 241.2838	0.9999	2.5
^51^V	0∼50 *µ*g·L^−1^	*f* (*x*) = 14661.8418*x* + 64.3682	0.9999	5.0
^60^Ni	0∼100 *µ*g·L^−1^	*f* (*x*) = 13025.5736*x* + 3519.1796	0.9998	10

**Table 7:** Results of specificity.

Elements		Blank	Sample blank	LOQ-1	LOQ-2	LOQ-3
Cd	Cd intensity (cps)	9	34	2282	2191	2293
	In intensity (cps)	243566	252744	237720	236767	237321
	Ratio (Cd/In)	0.000037	0.000135	0.009600	0.009254	0.009662

Pb	Pb intensity (cps)	7437	17912	68587	65069	66530
	Bi intensity (cps)	1728776	1729057	1628737	1626974	1633924
	Ratio (Pb/Bi)	0.004302	0.010359	0.042111	0.039994	0.040718

As	As intensity (cps)	7	9	1519	1498	1534
	Y intensity (cps)	162544	167769	158447	155792	157899
	Ratio (As/Y)	0.000043	0.000054	0.009587	0.009615	0.009715

Hg	Hg intensity (cps)	914	814	46330	45671	45876
	Bi intensity (cps)	1728776	1729057	1628737	1626974	1633924
	Ratio (Hg/Bi)	0.000529	0.000471	0.028445	0.028071	0.028077

Co	Co intensity (cps)	239	225	111883	108341	110411
	Y intensity (cps)	162544	167769	158447	155792	157899
	Ratio (Co/Y)	0.001470	0.001341	0.706123	0.695421	0.699251

V	V intensity (cps)	78	44	76045	74348	75912
	Y intensity (cps)	162544	167769	158447	155792	157899
	Ratio (V/Y)	0.000480	0.000262	0.479940	0.477226	0.480763

Ni	Ni intensity (cps)	3364	5208	129662	129676	130188
	Y intensity (cps)	162544	167769	158447	155792	157899
	Ratio (Ni/Y)	0.020696	0.031043	0.818330	0.832366	0.824502

**Table 8:** Accuracy of 7 elements in rosuvastatin calcium tablets.

Analyte	Added (*µ*g·L^−1^)	Found (*µ*g·L^−1^)	Recovery (%)
Cd	0.5 (50%)	0.506, 0.506, 0.518	100.4, 100.4, 102.8
	1 (100%)	1.002, 1.020, 0.977	99.8, 101.6, 97.3
	1.5 (150%)	1.501, 1.486, 1.488	99.8, 98.8, 98.9
	Average	—	99.8

Pb	0.5 (50%)	0.545, 0.544, 0.545	101.4, 101.2, 101.4
	1 (100%)	1.056, 1.074, 1.054	101.8, 103.6, 101.6
	1.5 (150%)	1.582, 1.554, 1.584	102.9, 101.1, 103.1
	Average	—	102.0

As	1.5 (50%)	1.427, 1.425, 1.455	94.6, 94.5, 96.5
	3 (100%)	2.853, 2.907, 2.845	94.8, 96.6, 94.6
	4.5 (150%)	4.280, 4.281, 4.253	94.9, 95.0, 94.3
	Average	—	95.1

Hg	3 (50%)	2.753, 2.764, 2.782	91.8, 92.1, 92.7
	6 (100%)	5.616, 5.581, 5.532	93.6, 93.0, 92.2
	9 (150%)	8.455, 8.383, 8.428	93.9, 93.1, 93.6
	Average	—	92.9

Co	5 (50%)	4.877, 4.907, 4.937	96.3, 96.9, 97.5
	10 (100%)	9.721, 9.707, 9.601	96.6, 96.4, 95.4
	15 (150%)	14.455, 14.432, 14.316	96.0, 95.8, 95.0
	Average	—	96.2

V	10 (50%)	10.234, 10.169, 10.292	97.6, 97.0, 98.2
	20 (100%)	20.045, 19.918, 19.755	97.9, 97.2, 96.4
	30 (150%)	29.398, 29.376, 29.304	96.4, 96.3, 96.1
	Average	—	97.0

Ni	20 (50%)	19.372, 19.422, 19.648	96.0, 96.3, 97.4
	40 (100%)	38.757, 38.859, 38.380	96.5, 96.7, 95.5
	60 (150%)	57.217, 57.290, 56.764	95.1, 95.2, 94.3
	Average	—	95.9

The concentration of Cd was 0.002 *µ*g·L^−1^, Pb was 0.019 *µ*g·L^−1^, As was 0.0038 *µ*g·L^−1^, Co was 0.031 *µ*g·L^−1^, V was 0.2354 *µ*g·L^−1^, and Ni was 0.0822 *µ*g·L^−1^.

**Table 9:** Precision of 7 elements in rosuvastatin calcium tablets.

Matrix	Recovery (%)						
	Cd	Pb	As	Hg	Co	V	Ni
Interday	99.8	100.9	95.1	95.6	97.1	99.1	97.1
	99.0	101.8	94.8	93.6	96.6	97.9	96.5
	101.5	103.6	96.6	93.0	96.4	97.2	96.7
	97.3	101.6	94.6	92.2	95.4	96.4	95.5
	96.9	102.1	95.3	91.3	95.3	98.3	96.0
	100.4	101.2	96.2	90.2	96.5	96.3	95.8

Intraday	99.7	99.8	93.3	95.6	96.2	97.2	96.8
	98.9	100.1	95.6	93.3	94.9	97.6	95.9
	100.2	105.3	94.6	92.8	95.7	98.0	94.7
	101.1	102.9	93.3	91.9	93.9	96.9	96.3
	99.7	104.5	95.8	94.6	96.1	94.9	96.8
	98.6	99.9	96.0	94.3	95.6	95.6	95.9

RSD (%)	1.4	1.8	1.1	1.8	0.9	1.2	0.7

## Data Availability

The data used to support the findings of the study are available from the corresponding author upon request.

## Supplementary Materials

Supplementary Materials ICP-MS was tuned before use. Tune report that contained parameters of the instrument was displayed in supplementary file.
