# Supplementary material for: Development and Validation of Analytical Procedure for Elemental Impurities in Rosuvastatin Calcium Tablets by ICP-MS and Microwave Digestion
Source: J Anal Methods Chem. 2024 Mar 26;2024:9952318. doi: 10.1155/2024/9952318 (PMC10987250; doi:10.1155/2024/9952318)
Supplement: Supplementary Materials — ICP-MS was tuned before use. Tune report that contained parameters of the instrument was displayed in supplementary file. [file 9952318.f1.pdf]

Autotune Report

QTEGRA

System

Start time:	2019-08-08 14:55:04
Instrument:	iCAP RQ
User:	942AI0075\fujuanGMP
Template:	SourceTune High Matrix
Instrument Serial Number:	RQ01490
Solution:	No solution specified
End time:	2019-08-08 15:04:55
Result:	The autotuning was successful.

Intensity Changes

Analyte	Original result	Tuned result
115In	1020837	1026176
140Ce	1060561	1104539
140Ce.16O	21614	20603
7Li	315229	289008
59Co	611814	580642
238U	1401504	1635416
140Ce.16O/140Ce	0.0204	0.0187

Control Changes

Control	Unit	Original value	Tuned value
Nebulizer Flow	[l/min]	1.01	0.9964
Torch Horizontal Position	[mm]	0	0
Torch Vertical Position	[mm]	0.75	0.77
Extraction Lens 2	[V]	-166	-200.7
CCT Focus Lens	[V]	3.6	3.6

Autotune Report

Graphical Results

Stage name: Torch position
Control: Torch Horizontal Position
Analyte: 115In

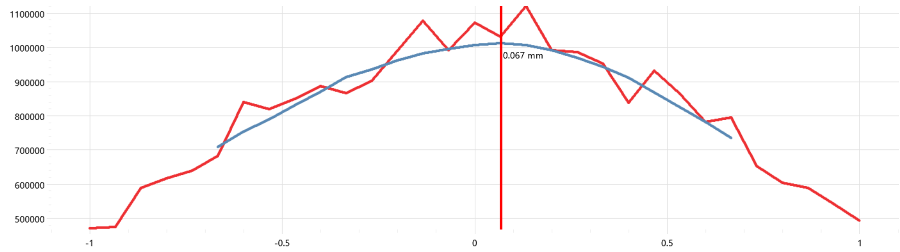

Stage name: Torch position
Control: Torch Vertical Position
Analyte: 115In

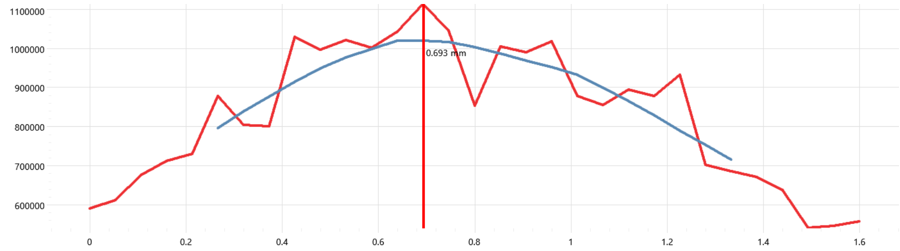

Stage name: Nebulizer (2.5% CeO)
Control: Nebulizer Flow
Analyte: 115In

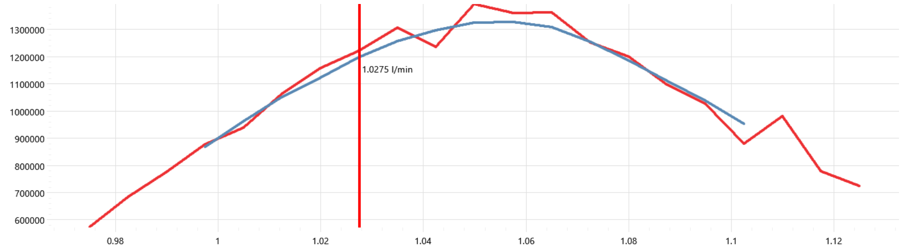

Stage name: Nebulizer (2.5% CeO)
Control: Nebulizer Flow
Analyte: 140Ce.16O/140Ce

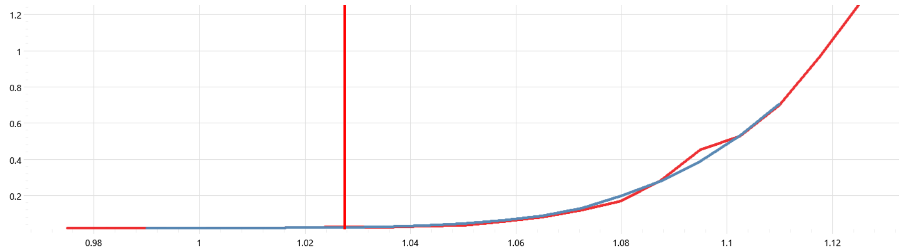

Stage name: Torch position again
Control: Torch Horizontal Position

Autotune Report

Analyte: 115In

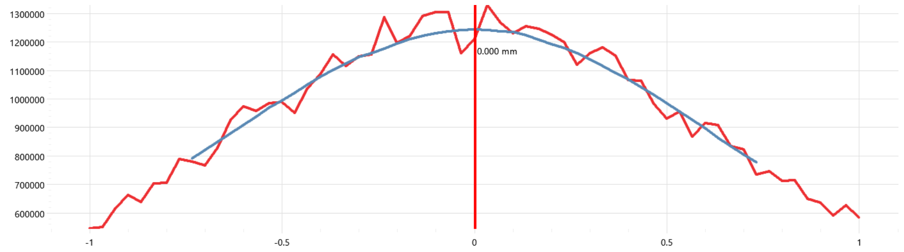

Stage name: Torch position again
Control: Torch Vertical Position
Analyte: 115In

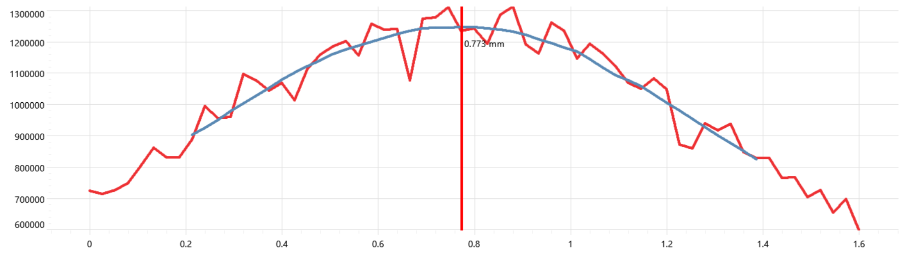

Stage name: Extraction Lens 2
Control: Extraction Lens 2
Analyte: 115In

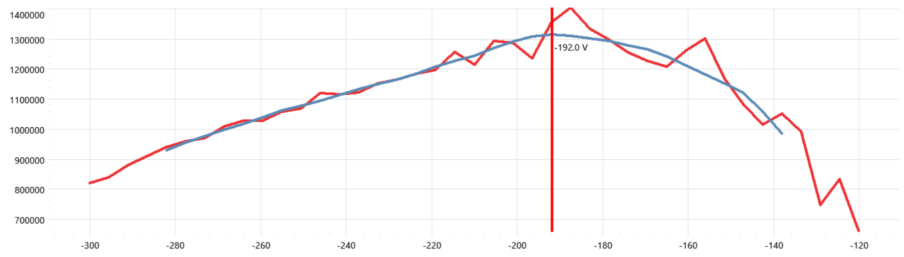

Stage name: CCT Focus Lens
Control: CCT Focus Lens
Analyte: 238U

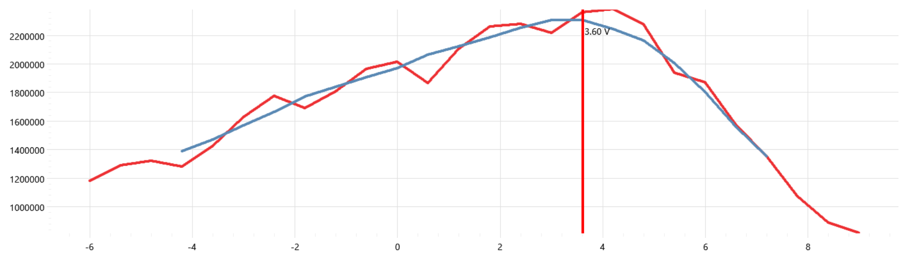

Stage name: Nebulizer (1.9 % CeO)
Control: Nebulizer Flow
Analyte: 115In

Autotune Report

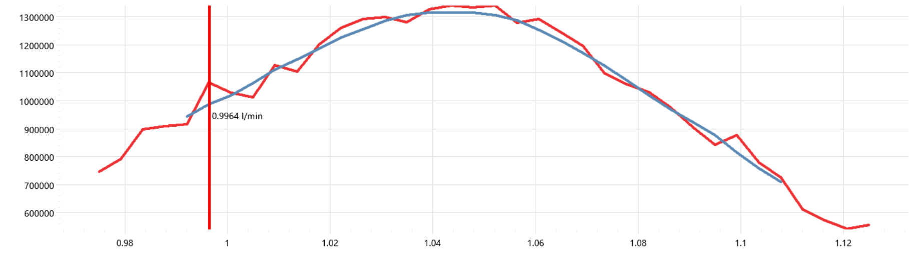

Stage name: Nebulizer (1.9 % CeO)
Control: Nebulizer Flow
Analyte: 140Ce.16O/140Ce

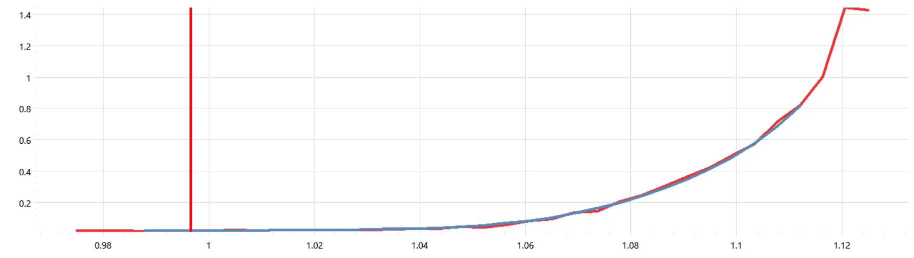

Stage name: Extraction Lens 2 @ final Plasma Temperature
Control: Extraction Lens 2
Analyte: 59Co

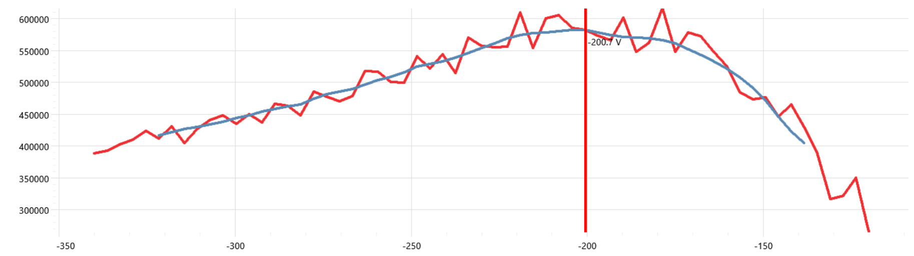

Stage name: CCT Focus Lens @ final Plasma Temperature
Control: CCT Focus Lens
Analyte: 7Li

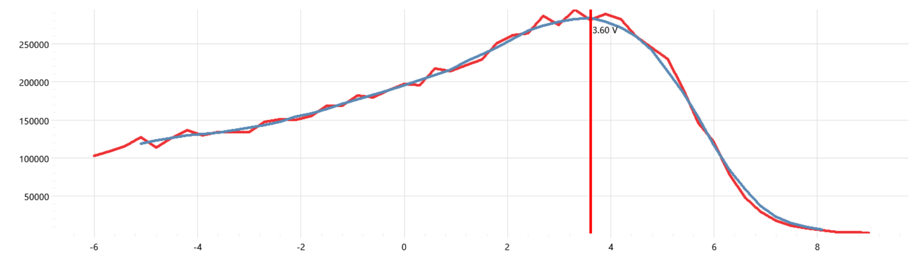

Stage name: CCT Focus Lens @ final Plasma Temperature
Control: CCT Focus Lens
Analyte: 238U

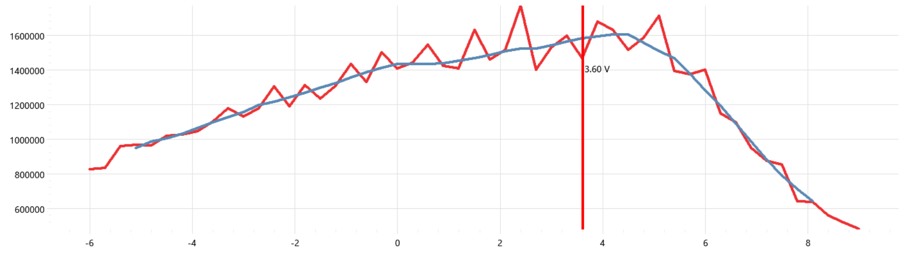
1600000
1400000
1200000
1000000
800000
600000
3.60 V
-6
-4
-2
0
2
4
6
8

Autotune Report

QTEGRA

Tune Settings

Parameter	Value
Additional Gas Flow 1	0
Additional Gas Flow 2	0
Additional Gas Flow 3	0
CCT Entry Lens	-106
Angular Deflection	-382.4
Deflection Entry Lens	-35
Extraction Lens 1 Polarity	0
Extraction Lens 1 Negative	0
Extraction Lens 1 Positive	0
Spray Chamber Temperature	2.7
Peristaltic Pump Speed	40
Cool Flow	14
Sampling Depth	5
Plasma Power	1550
Auxilliary Flow	0.8
Nebulizer Flow	0.996428571428573
Torch Horizontal Position	-1.38777878078145E-17
Torch Vertical Position	0.773333333333333
Extraction Lens 2	-200.666666666666
CCT Focus Lens	3.6
CCT Bias	-2
CCT Exit Lens	-160
Focus Lens	17.4
D1 Lens	-195.2
D2 Lens	-80
Quad Entry Lens	-32.2
Pole Bias	-1
CCT1 Flow	0
CCT1 Shut-Off Valve	0
Virtual CCT Mass to Dac Factor	130
Virtual CCT Mass to Dac Offset	-167.5
Virtual CCT Mass parameter b	0.65
Virtual CCT Mass Maximum Dac Limit Set	4095
